# Pectocin M1 (PcaM1) Inhibits *Escherichia coli* Cell Growth and Peptidoglycan Biosynthesis through Periplasmic Expression

**DOI:** 10.3390/antibiotics5040036

**Published:** 2016-10-08

**Authors:** Dimitri Chérier, Sean Giacomucci, Delphine Patin, Ahmed Bouhss, Thierry Touzé, Didier Blanot, Dominique Mengin-Lecreulx, Hélène Barreteau

**Affiliations:** Institute for Integrative Biology of the Cell (I2BC), CEA, CNRS, Univ Paris-Sud, Université Paris-Saclay, Gif-sur-Yvette 91198, France; dimitri.cherier@i2bc.paris-saclay.fr (D.C.); sean.giacomucci@umontreal.ca (S.G.); delphine.patin@i2bc.paris-saclay.fr (D.P.); ahmed.bouhss@univ-evry.fr (A.B.); thierry.touze@i2bc.paris-saclay.fr (T.T.); didier.blanot@gmail.com (D.B.); dominique.mengin-lecreulx@i2bc.paris-saclay.fr (D.M.-L.)

**Keywords:** pectocin M1, peptidoglycan, bacteriocin, colicin, periplasmic expression

## Abstract

Colicins are bacterial toxins produced by some *Escherichia coli* strains. They exhibit either enzymatic or pore-forming activity towards a very limited number of bacterial species, due to the high specificity of their reception and translocation systems. Yet, we succeeded in making the colicin M homologue from *Pectobacterium carotovorum*, pectocin M1 (PcaM1), capable of inhibiting *E. coli* cell growth by bypassing these reception and translocation steps. This goal was achieved through periplasmic expression of this pectocin. Indeed, when appropriately addressed to the periplasm of *E. coli*, this pectocin could exert its deleterious effects, i.e., the enzymatic degradation of the peptidoglycan lipid II precursor, which resulted in the arrest of the biosynthesis of this essential cell wall polymer, dramatic morphological changes and, ultimately, cell lysis. This result leads to the conclusion that colicin M and its various orthologues constitute powerful antibacterial molecules able to kill any kind of bacterium, once they can reach their lipid II target. They thus have to be seriously considered as promising alternatives to antibiotics.

## 1. Introduction

Colicins are bacterial toxins produced by some *Escherichia coli* strains in order to kill susceptible strains of *E. coli* and related species. Among the ca*.* twenty colicins identified to date [1], colicin M (ColM) is unique as it is the only one interfering with bacterial peptidoglycan biosynthesis. ColM acts by cleavage of the last precursor of this essential metabolic pathway, thereby causing cell death. Indeed, it was shown ten years ago, both in vivo and in vitro, that ColM was an enzyme catalyzing specifically the hydrolysis of the peptidoglycan lipid intermediate C_55_-PP-MurNAc(pentapeptide)-GlcNAc (lipid II), by cleaving between the prenyl chain (C_55_-) and the pyrophosphoryl group of this precursor [2]. The two reaction products, undecaprenol and 1-PP-MurNAc(pentapeptide)-GlcNAc, that do not normally exist in *E. coli* growing cells, were accumulated in the ColM-treated cells. These products cannot be reused into peptidoglycan metabolism or be recycled, thereby leading to cell lysis.

ColM develops its bacteriolytic activity in three sequential steps deeply linked to its structural organization in three domains and parasitizes proteins from the targeted cell to enter the periplasm. Accordingly, the ColM central domain first binds to an outer membrane receptor for siderophores, FhuA [3]. Subsequently, it is translocated towards the periplasm by using the proton-motive force, through the interaction of its N-terminal domain with the TonB machinery system comprising the TonB, ExbB and ExbD proteins [4]. Finally, once shuttled into the periplasm of the targeted cell, ColM is maturated by the ubiquitous chaperone protein FkpA, so that its C-terminal domain can display its cytotoxic effect on lipid II [5,6].

As this is the case for many bacteriocins, ColM-producing cells are protected against the lethal effect of their own colicin by the concomitant synthesis of an immunity protein, named ImM (or Cmi). Although the latter protein has been structurally characterized [7,8], the mechanism of protection of the bacterial cell from lysis is still unknown. However, non-producing cells can also protect themselves against the action of ColM by mutation or deletion of genes encoding proteins involved in ColM reception/translocation/maturation processes [9,10].

During the last few years, several ColM orthologues have been identified, by sequence alignments of their C-terminal domains, in some other bacterial genera, such as *Pseudomonas*, *Burkholderia* and *Pectobacterium* species, and several of them have been both biochemically and structurally characterized [11,12,13,14,15]. All of the purified ColM orthologues displayed the same enzymatic activity of cleavage of lipid II and a bacteriolytic (or at least bacteriostatic) activity [11,12]. Yet, despite their similarities in terms of enzymatic activity, these orthologues did not share the same 3D structure, especially in their N-terminal domains. On the one hand, ColM shared a very compact structure with PaeM and syringacin M (also named PsyM), i.e., its orthologues from *Pseudomonas aeruginosa* and *Pseudomonas syringae*, respectively, where the usual three structural domains did not form distinct entities [12,13]. On the other hand, the structure of pectocin M2 from *Pectobacterium* *carotovorum* and probably also that of pectocin M1, since it has been strongly suggested that both pectocins M1 and M2 used the same outer membrane receptor on *Pectobacterium* spp. [14], presented an atypical translocation domain, where the helical receptor-domain and unstructured N-terminus were replaced by a single globular plant-like [2Fe-2S] ferredoxin domain, directly connected to the cytotoxic one through a small α-helix linker [15]. Moreover, while numerous receptors for colicins and closely-related pyocins were Tol- or TonB-dependent, these ferredoxin-containing pectocins presumably used a bacterial ferredoxin uptake mechanism to cross the outer membrane, without any evidence for Tol or TonB complex requirements [15].

The cytotoxicity of all of these ColM-like bacteriocins has been previously demonstrated to be pointed towards a limited number of bacterial species. The specificity of their receptor-binding and translocation domains presumably prevents them from targeting a broad range of bacteria. It thus seems that, if reaching the target constitutes a crucial step for the colicin cytotoxicity, it is also the limiting one. Yet, ways allowing the bypass of this specific and limiting step do exist. Indeed, the use of an osmotic shock was shown to be efficient to make extracellular proteins enter the periplasm of Gram-negative bacteria without loss of cell integrity [16,17]. Another efficient means to send proteins into the periplasm of a target cell was the use of fusion proteins between the bacteriocin of interest and a signal sequence. This strategy was particularly used to study the pore formation by colicin A [18,19]. According to the latter methodology, we describe in the present paper how we managed to evaluate the antibacterial activity of pectocin M1 (that will be named PcaM1 throughout this work, following the nomenclature we previously used [11]) and some of its variants on *E. coli* cells by triggering their periplasmic expression. We show that when addressed to the periplasm of *E. coli*, the PcaM1 protein, as well as its isolated activity domain could catalyze the degradation of the peptidoglycan lipid II precursor, which leads to the arrest of the biosynthesis of this essential cell wall component and, consequently, to cell lysis. It is also shown that, contrary to ColM, this pectocin does not depend on the presence of the host FkpA chaperone protein to exert its deleterious toxic effects.

## 2. Results

### 2.1. Design, Production and Purification of Wild-Type and Mutant PcaM1

The gene encoding PcaM1 was amplified from the *P. carotovorum* ssp*. carotovorum* strain PC1 and cloned into a pET expression vector allowing the production of a C-terminal His_6_-tagged protein. The catalytic point mutant (D222A) in which Asp222 is replaced by Ala, which was previously reported to be devoid of cytotoxic activity [14], was also produced as a C-terminal His_6_-tagged protein.

According to SDS-PAGE analysis of the crude extracts, the best recovery yields of both wild-type and mutant proteins were obtained following overnight IPTG-induced expression at 22 °C. Thereafter, the proteins were purified by affinity chromatography with an overall yield of about 10 mg per liter of culture, and their purity, as judged by SDS-PAGE, was about 95%. Subsequent gel filtration analysis showed that both wild-type and mutant forms of PcaM1 mainly eluted as monomers. No protein aggregates were observed, confirming that the proteins were homogeneous (Supplementary Materials Figure S1). As previously described, both wild-type and mutant purified recombinant proteins had a red-brown color and displayed absorption spectrum maxima at 329, 423 and 467 nm [14]. It should be noted that when protein samples were not boiled before SDS-PAGE analysis, two slightly differently-migrating protein bands of the expected molecular mass for PcaM1 were observed (Supplementary Materials Figure S1). The fast migrating species could represent a more compact and, thus, a more SDS-resistant form of the protein. It is tempting to link this observation to the two protein populations (compact and extended forms) that have been previously revealed by SAXS experiments on pectocin M2 by Grinter and collaborators [15]. MALDI-TOF mass spectrometry analyses of the wild-type PcaM1 revealed only one major peak at *m/z* 30,266 Da for the [M + H]^+^ ion, which is in agreement with the theoretical mass of 30,401 Da calculated for the His_6_-tagged protein and with the loss of the N-terminal methionine residue (Supplementary Materials Figure S2).

### 2.2. Enzymatic Properties of PcaM1

**Figure 1:**
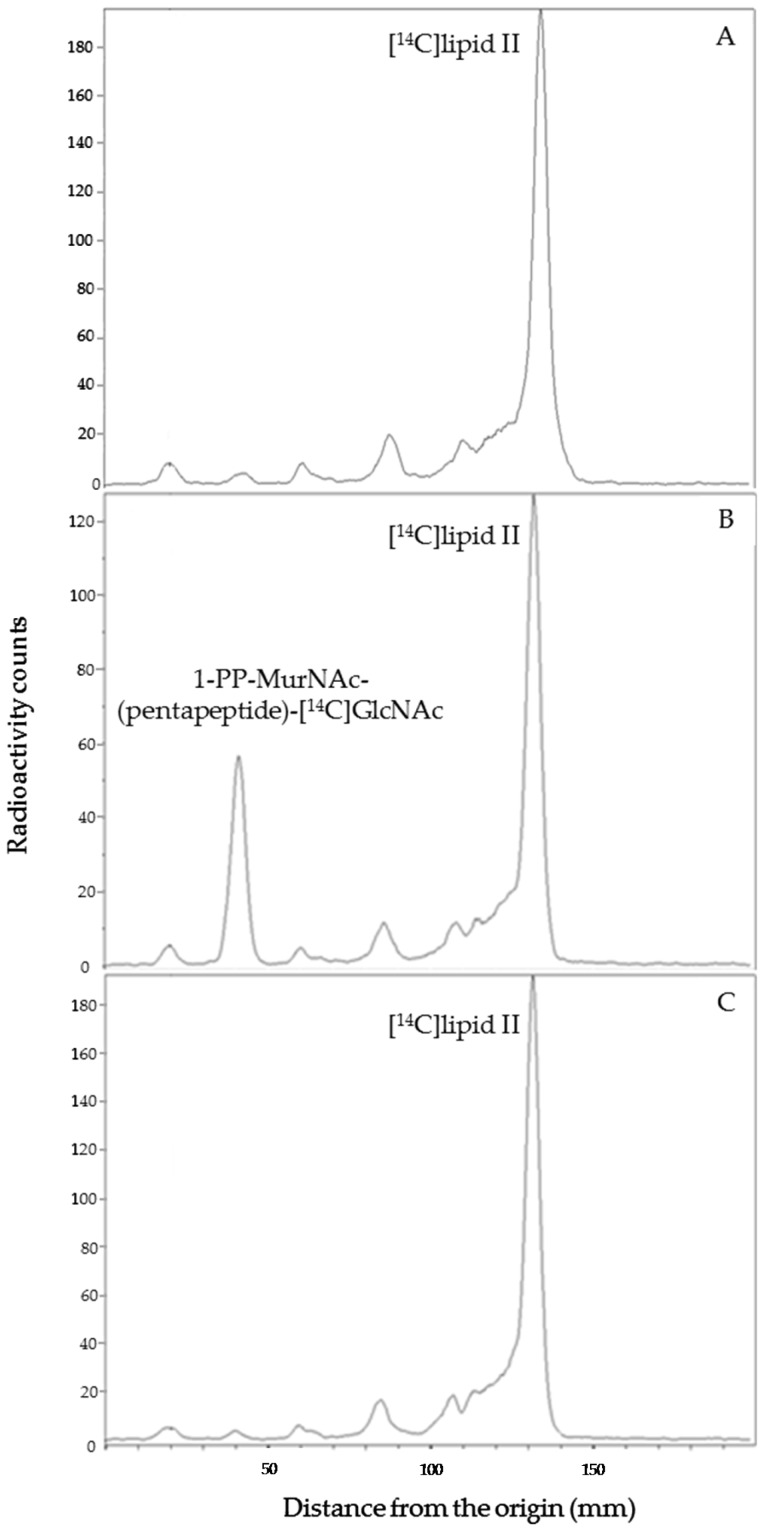
In vitro degradation of lipid II by PcaM1. Conditions that result in ca*.* 20% hydrolysis of radiolabeled lipid II were used. Purified [^14^C]lipid II radiolabeled in the GlcNAc moiety (140 Bq) was incubated without (**A**) or with 1.2 µg of wild-type (**B**) or D222A mutant (**C**) PcaM1. The substrate and reaction product were separated by TLC (solvent system: 1-propanol/ammonium hydroxide/water; 6:3:1; v/v/v), and the corresponding spots were detected with a radioactivity scanner, as detailed in the text (*R_f_* = 0.7 and 0.3 for lipid II and 1-PP-MurNAc(pentapeptide)-GlcNAc, respectively).

Pure wild-type PcaM1 was tested for its capacity to hydrolyze the peptidoglycan intermediate lipid II in vitro, as previously shown for ColM and its homologues from *Pseudomonas* species [2,11]. The peptidoglycan composition of the genus *Pectobacterium* is not known to date, but as this genus belongs to the *Enterobacteriaceae*, a classical diaminopimelic acid (A_2_pm)-containing peptidoglycan structure is likely present in this bacterium [20]. Therefore, the corresponding lipid II was synthesized by using *E. coli* MraY and MurG enzymes to be used as a substrate in our assays [2]. PcaM1 was able to convert the radiolabeled [^14^C]lipid II substrate to a reaction product migrating with a lower *R_f_* (0.3 vs*.* 0.7 for lipid II) on TLC plates (Figure 1A,B), confirming the enzymatic nature of this protein and its similarity with ColM and the *Pseudomonas* orthologues [2,11].

Under the standard assay conditions used, the specific activity of PcaM1 was 0.53 nmol/min/mg of protein, which is quite similar to that previously determined for ColM (0.4 nmol/min/mg) [2]. The *K*_m_ value of wild-type PcaM1 for *meso*-A_2_pm-containing lipid II was determined to be 55 µM, which is also in the usual range of other ColM-orthologues [11].

The ability of the D222A PcaM1 mutant to hydrolyze lipid II was also tested in the same conditions. As expected, this catalytic point mutant did not exhibit any in vitro activity of degradation of the lipid II (Figure 1C), even when high amounts of proteins were used (up to 15 µg per assay).

### 2.3. Cytotoxicity of PcaM1 and Its Variants

**Figure 2:**
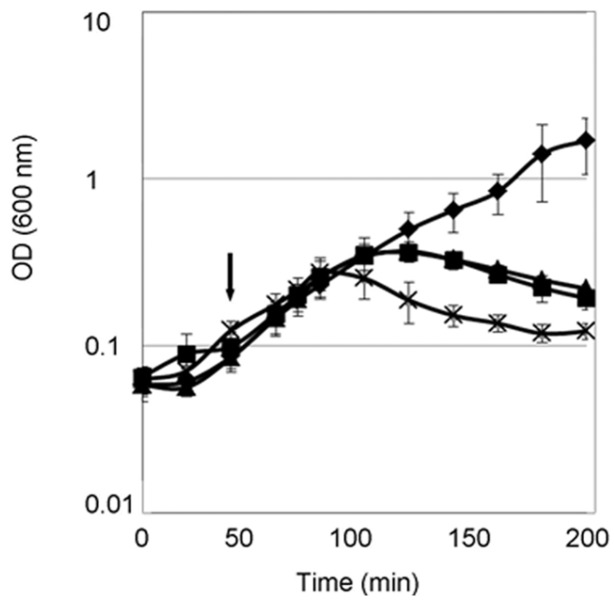
Effects of the periplasmic expression of PcaM1 variants on the growth of *E. coli*. FB8 cells were grown at 37 °C in 2YT medium and the expression of the PcaM1 variants was induced by the addition of anhydrotetracycline (arrow). Growth curves observed for the FB8 strain carrying the pASK-IBA4 empty vector or pASK plasmids expressing the wild-type PcaM1, the D222A mutant or the isolated activity domain (Δ1-107 PcaM1) are shown by diamonds, squares, triangles and crosses, respectively. Four independent experiments were performed for each strain.

Purified PcaM1 was tested on agar plates for its cytotoxic activity against various wild-type *E. coli* strains (DH5α, BW25113, FB8), and it was found to be totally inactive, as expected. Then, in order to bypass the receptor and translocation machineries, the potential cytotoxicity of PcaM1 was highlighted on the *E. coli* FB8 strain by using the pASK-IBA4 vector, which allowed a controlled expression and subsequent export towards the periplasm of this bacteriocin thanks to its fusion to the OmpA protein signal sequence. Indeed, the addition of anhydrotetracycline triggered the expression of PcaM1 and induced a rapid lysis of *E. coli* FB8 cells (Figure 2). To the best of our knowledge, this is the first time that a bypass of the species specificity following periplasmic expression was described for a member of the ColM family, whose members have been reported to possess a narrow antibacterial spectrum.

We then designed two variants of PcaM1: the same catalytic point mutant (D222A), which had been expressed in the pET vector, and a truncated form deleted of the 107 first N-terminal amino-acid residues (which corresponds to the isolated activity domain). Both variants were tested for their potential toxicity in the *E. coli* FB8 strain background. An identical lytic phenotype was observed following induction of the periplasmic expression of these two proteins (Figure 2).

Such a phenomenon was somewhat expected for the strain expressing the truncated form of PcaM1, considering that isolated ColM and PaeM catalytic domains generated by protein dissection experiments were previously demonstrated to be enzymatically active [13,21]. In contrast, data obtained with the strain expressing the PcaM1 D222A mutant were more surprising, as it was previously shown that the mutation (D226N) of the corresponding catalytic residue of ColM completely abolished the enzymatic activity and toxicity of the latter colicin when exported into the periplasm [22].

As the P_tet_ promoter from the pASK-IBA4 vector is known to be a reasonably strong promoter, we checked whether the lytic phenotype observed for strains expressing wild-type and mutant PcaM1 proteins was not due to a non-specific protein overexpression effect, but rather to the intrinsic toxicity of the PcaM1 activity. Decreasing the concentration of the inducer anhydrotetracycline (30–200 ng/mL range) and, consequently, of PcaM1 expression led to the same growth defect and lytic behavior for both strains (Supplementary Materials Figure S3). Then, to confirm that the decrease of the optical density of the cultures indeed reflected a loss of viability and cell lysis, the experiments of growth monitoring were repeated at the anhydrotetracycline concentration of 200 ng/mL, and colony forming unit counting was performed in parallel to determine the survival ratio in each case (Supplementary Materials Figure S4). These experiments revealed that cell lethality occurred in both cases, as we observed a three-log decrease in CFU for the strain expressing wild-type PcaM1 ca*.* 30 min after the anhydrotetracycline was added, but only a two-log decrease in CFU after the same time for the strain expressing the D222A PcaM1 mutant, thus showing a less pronounced loss of viability effect for the mutant.

Our data thus demonstrated that the PcaM1 and its derivatives were able to bypass the biological “partners” needed for internalization of a regular exogenous bacteriocin, that is the receptor and translocation machineries. Then, to address a possible role of the maturation chaperone protein FkpA in the activity of PcaM1, the same type of experiment was performed in the *E. coli* BW25113 Δ*fkpA* mutant strain (Supplementary Materials Figure S5). Unlike ColM, whose toxicity of the full-length protein and isolated catalytic domain was found to be FkpA-dependent and independent, respectively, a lytic phenotype was similarly observed here with all of the PcaM1 variants in a Δ*fkpA* genetic background. PcaM1 and its two variants thus did not need a maturation process by FkpA to be active in vivo, once delivered in the *E. coli* periplasm. This result was quite interesting, as only the truncated form of ColM was reported to date to be FkpA-independent [22].

### 2.4. Consequences of the Periplasmic Expression of PcaM1 and Its Derivatives on Peptidoglycan Metabolism in E. coli

**Figure 3:**
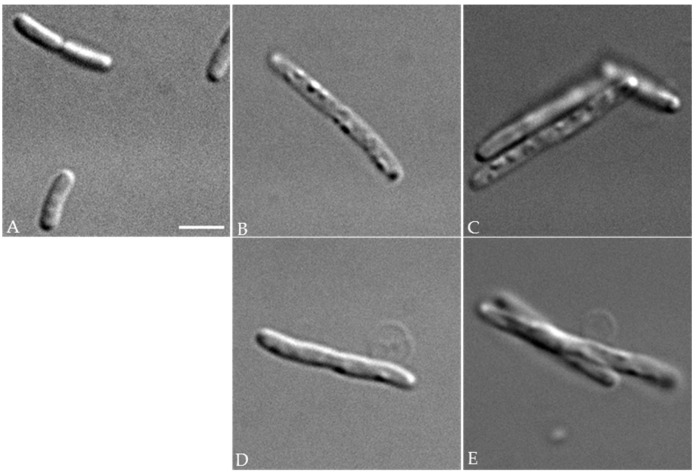
Morphology of *E. coli* strains. Optical micrographs of exponentially-growing control FB8 cells (**A**) and of cells expressing either the wild-type (**B**,**D**) or the D222A mutant (**C**,**E**) PcaM1. As compared to control cells, those expressing either form of PcaM1 in the periplasm appeared elongated, presented swellings on their surface (**B**,**C**), and some of them were blebbing (**D**,**E**). The scale bar shown is 3 µm.

To better understand the effects of the periplasmic expression of these enzymes in *E. coli*, microscopic examination of FB8 cells expressing wild-type and mutant forms of PcaM1 was performed. As compared to control cells that were relatively small and presented a smooth surface (Figure 3A), wild-type, as well as mutant PcaM1-expressing cells appeared elongated and displayed multiple swelling zones on their surface (Figure 3B–D,C–E, respectively). Some of these cells also exhibited amazing membrane protuberances (Figure 3D,E) that ultimately burst. These observations demonstrated that both wild-type and mutant forms of PcaM1 similarly affected *E. coli* cell growth and morphology, a phenotype that was consistent with a perturbation of their peptidoglycan metabolism.

**Figure 4:**
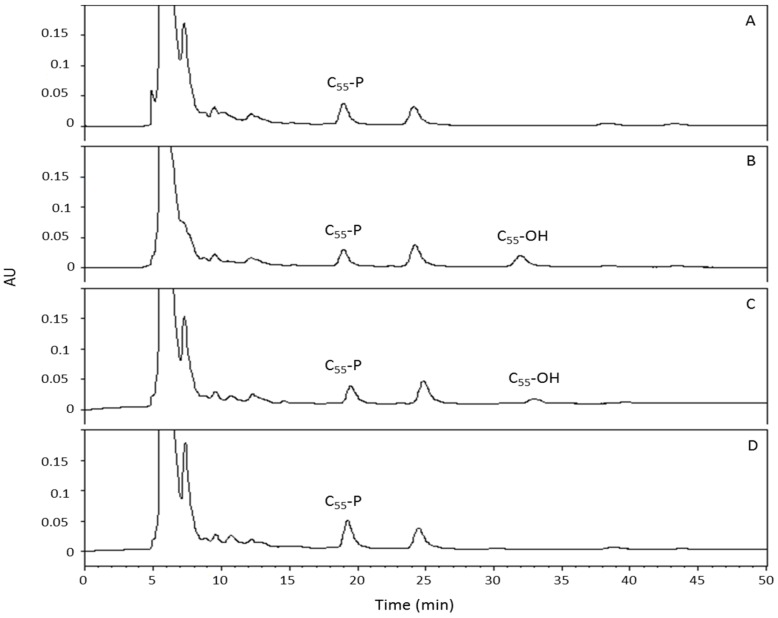
HPLC analysis of membrane extracts (Bligh and Dyer procedure) prepared from *E. coli* FB8 cells carrying either the empty pASK vector (**A**) or pASK plasmids expressing the wild-type PcaM1 (**B**), its isolated cytotoxic domain (**C**) or the D222A mutant (**D**), respectively. Cells were grown exponentially in 2YT medium, and PcaM1 variants’ expression was induced at OD_600_ = 0.2 by the addition of anhydrotetracycline. Cells were harvested just before the onset of lysis and treated by sonication. Membrane extracts were prepared and isoprenoids extracted and analyzed by HPLC as described in the text.

To test this hypothesis further, the pool levels of the carrier lipid undecaprenyl phosphate (C_55_-P) and of its two derivatives undecaprenyl pyrophosphate (C_55_-PP) and undecaprenol (C_55_-OH) were determined in membranes of *E. coli* cells expressing the three PcaM1 variants, using previously-described procedures [23]. Indeed, ColM is known to provoke the cleavage of lipid II into C_55_-OH, thereby blocking the recycling of the carrier lipid C_55_-P and peptidoglycan synthesis [2]. Following the expression of PcaM1 and its resulting growth defect, a pool of the isoprenoid C_55_-OH was thus expected to appear. The results of the isoprenoid HPLC analysis and quantification are presented in Figure 4 and Table 1.

These experiments allowed us to detect the appearance and accumulation of C_55_-OH in membranes of *E. coli* cells expressing wild-type and truncated forms of PcaM1 (Figure 4), which was expected considering the lytic effect of this bacteriocin in liquid culture conditions and was reminiscent of previous data observed with ColM [2,23]. This C_55_-OH pool represented between one third and a half of the total isoprenoid pool in both *E. coli* strains. Another interesting trait was the total isoprenoid content that remained generally quite similar, irrespective of the strain considered (Table 1). In both the full-length and truncated PcaM1-expressing strains, this effect is likely due to the fact that the C_55_-PP pool was depleted to maintain the C_55_-P pool at a standard level of about 75 nmol/g of dry cell weight, as previously described for *E. coli* [23].

However, the *E. coli* strain expressing the D222A mutant form of PcaM1 did not show the same pattern of isoprenoids. Surprisingly indeed, although this mutant also provoked *E. coli* cell lysis in liquid culture conditions, membranes of the latter strain did not contain any detectable C_55_-OH. Here too, the pool of C_55_-PP was significantly depleted, but a ca*.* 60% increase of the C_55_-P pool was observed in these conditions. As will be discussed later, these findings suggested that the D222A enzymatically-inactive mutant also inhibited the same step of the peptidoglycan synthesis pathway, without displaying any lipid II-degrading activity.

**Figure 5:**
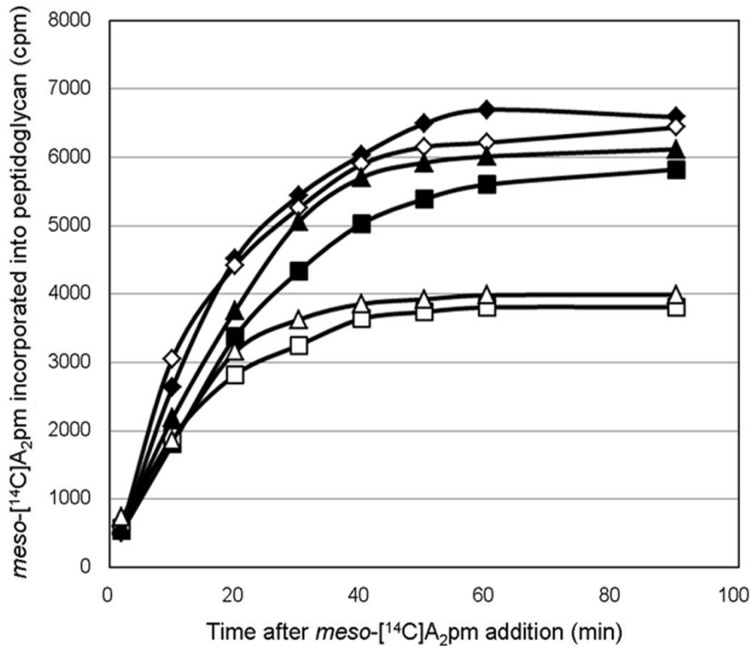
Effect of the periplasmic expression of wild-type and mutant forms of PcaM1 on *meso*-[^14^C]A_2_pm incorporation into peptidoglycan. Cells were grown in M63-glucose minimum medium supplemented with lysine, threonine and methionine. When the OD_600_ reached 0.2, anhydrotetracycline was added, or not, and *meso*-[^14^C]A_2_pm was added 10 min thereafter. The incorporation of radioactivity into the peptidoglycan (trichloroacetic acid (TCA)-insoluble material) was then followed as described in the text. Symbols: diamonds, strain carrying the pASK-IBA4 empty vector; squares and triangles, strains expressing the wild-type and D222A mutant PcaM1, respectively. Open and closed symbols represent cultures treated or not with anhydrotetracycline, respectively.

To unambiguously demonstrate that the observed cell lysis was the consequence of an arrest of peptidoglycan synthesis and to investigate why the strain expressing the catalytic mutant form of PcaM1 also lysed, cell labeling experiments were performed, in which radiolabeled *meso*-[^14^C]A_2_pm was used as a specific marker of peptidoglycan biosynthesis. To ensure a specific and optimal labeling of peptidoglycan, an *E. coli* FB8 Δ*lysA* mutant strain was used and grown in minimal medium conditions, as previously described [2,24]. In this way, we were able to follow the specific incorporation of *meso*-[^14^C]A_2_pm into the peptidoglycan macromolecule of *E. coli* cells expressing wild-type and mutant forms of PcaM1, compared to a control strain (Figure 5).

As shown in Figure 5, *meso*-[^14^C]A_2_pm was rapidly incorporated into the peptidoglycan polymer after its addition to the culture medium. When anhydrotetracycline was added, this incorporation was rapidly blocked in strains expressing either the wild-type or the D222A mutant form of PcaM1, but not in the control strain carrying the empty vector pASK-IBA4. This arrest of incorporation happened about 30 min after *meso*-[^14^C]A_2_pm addition to the culture medium. As observed in Figure 2, *E. coli* cell lysis started about 40 min after addition of anhydrotetracycline. The observed lytic phenotype was thus clearly correlated to an arrest of peptidoglycan synthesis.

We then investigated the cellular distribution of the radioactive material in cells expressing PcaM1 variants. Radioactivity counts recovered in the soluble and insoluble cell fractions from both strains are presented in Table 2, compared to control cells.

**Figure 6:**
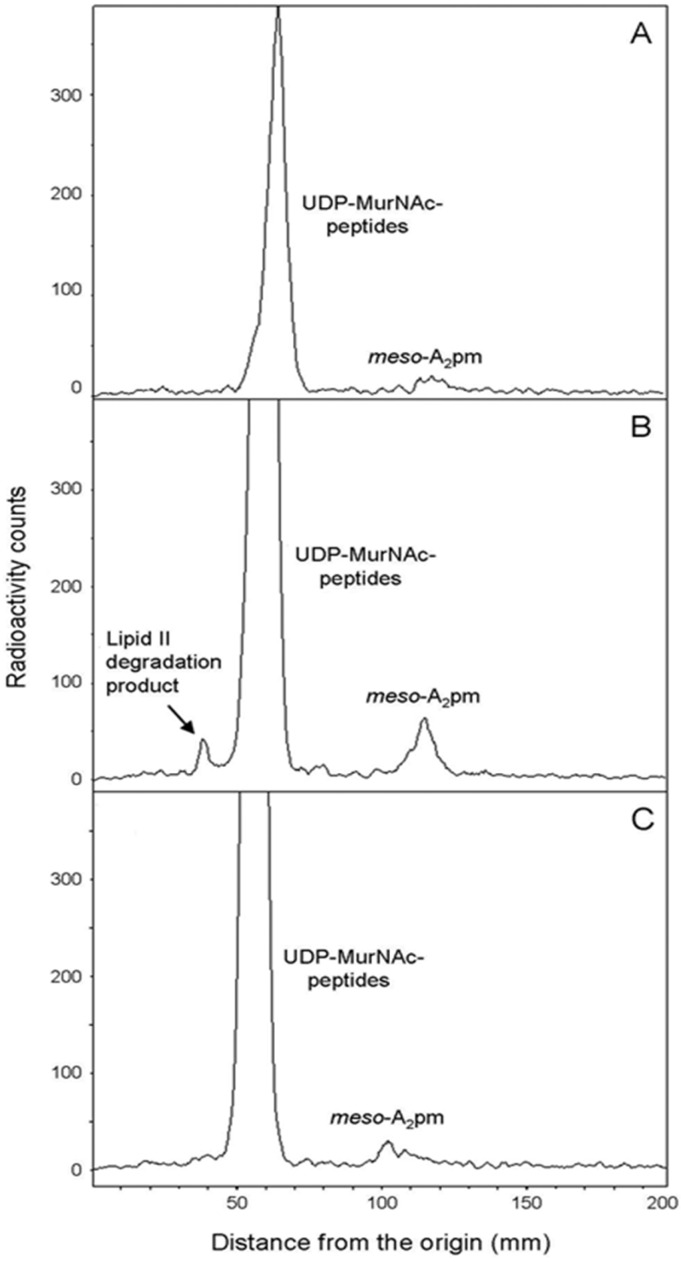
TLC analysis of the soluble fractions from *E. coli* FB8 *lysA* cells expressing (**B**,**C**) or not (**A**) the wild-type and D222A mutant forms of PcaM1, respectively. The peak corresponding to UDP-MurNAc-peptides was shown to contain essentially UDP-MurNAc-pentapeptide (>95%) and low amounts of UDP-MurNAc-tripeptide by HPLC analysis. A peak of radiolabeled lipid II degradation product 1-PP-MurNAc(pentapeptide)-GlcNAc was only observed in cells expressing the wild-type form of PcaM1.

These data first showed that the total radioactivity incorporated in the three *E. coli* strains was similar, ca*.* 300,000 counts per min in the typical experiment shown, indicating that the expression of the PcaM1 variants did not affect the *meso*-[^14^C]A_2_pm uptake. Expression of PcaM1 was shown to result in a 60% decrease of incorporated radioactivity in the insoluble fraction, which contains peptidoglycan (mainly) and lipids I and II intermediates (Table 2), consistent with our previous data shown in Figure 3. As a result, the “missing” radioactivity that has not been incorporated in the polymer was found to be accumulated in the soluble fraction, which is known to contain *meso*-[^14^C]A_2_pm and the peptidoglycan nucleotide precursors as the main labeled compounds. Chromatography (TLC) analysis of these fractions (Figure 6) confirmed the decrease of radioactivity counts incorporated in the polymer and the significant (four-fold) accumulation of the UDP-MurNAc-pentapeptide precursor as the main soluble radiolabeled compound, as expected for an inhibition of the membrane steps of this pathway. Moreover, an additional peak of radiolabeled compound was observed (*R_f_* of 0.3), specifically when soluble extracts from the strain expressing the wild-type PcaM1 were analyzed (Figure 6). This peak, which was also observed in ColM-treated cells, had been earlier identified as the lipid II-degradation product 1-PP-MurNAc(pentapeptide)-GlcNAc [2]. The absence of this peak in extracts from cells expressing the D222A variant was perfectly consistent with the absence of a detectable pool of C_55_-OH in these cells, as well as with the inactivity of this mutant protein.

## 3. Discussion

In this work, we focused our investigations on PcaM1, one of the two ColM-like orthologues produced by *P. carotovorum*. We first revisited the results previously published by Grinter and collaborators in 2012 by producing the wild-type and catalytic point mutant D222A PcaM1 proteins. We thus confirmed, on the one hand, the enzymatic activity of lipid II degradation by the wild-type PcaM1, whereas the mutant was inactive, and on the other hand, the absence of cytotoxic activity of PcaM1 preparation on *E. coli* cells, as no growth inhibition zone was detected when up to 57 µg of this protein were spotted on *E. coli* BW25113-inoculated agar plates.

Although PcaM1, as well as the other ColM orthologues [11] did not display any cytotoxic activity against *E. coli* cells, we recently showed that the application of an osmotic shock treatment to *E. coli* cells allowed the *P. aeruginosa* ColM orthologue (PaeM) to bypass the outer membrane reception and translocation steps, reach its lipid II target and thus exert its deleterious activity [13]. This allowed us to demonstrate that PaeM, as well as its isolated catalytic domain, was able to kill *E. coli* cells. It was the first example of a ColM-like protein capable of killing another bacterial species. 

Another way to get access to the *E. coli* periplasmic space was to fuse ColM to the OmpA protein signal sequence. In these conditions, the hybrid ColM was directly exported from the cytoplasm to the periplasm of the producing cells and was then demonstrated to be toxic [22]. Therefore, to check whether another ColM orthologue would be able to kill *E. coli* cells by this approach, we fused the PcaM1 to OmpA signal sequence and expressed the hybrid protein in *E. coli*. In this way, we visualized that PcaM1 was toxic for *E. coli* cells, as the controlled induction of its expression led to cell lysis. To the best of our knowledge, this is the first example of cell lysis due to periplasmic expression of a ColM-like orthologue. A hybrid protein obtained by fusion of the PcaM1 variant truncated of its 107 first amino acids to the OmpA signal sequence led to the same lytic phenotype, as also did the D222A point mutant PcaM1. That the expression of the isolated catalytic domain of PcaM1 yielded cell lysis in these conditions was somewhat expected, as this had been observed also with the catalytic domain of ColM [22]. However, the lytic effect exhibited by the D222A variant was much more surprising. Indeed, as described above, no enzymatic activity of in vitro degradation of lipid II was reported for this mutant protein. The Asp222 residue from PcaM1 corresponds to Asp226 in ColM, which has been previously demonstrated to be essential for the catalytic activity and, consequently, for the cytotoxicity of ColM [21]. Moreover, it was previously shown that the D226N mutant of ColM did not display any toxic activity when exported to the periplasm [22]. The lytic phenotype of the *E. coli* strain expressing the D222A PcaM1 mutant was thus intriguing. Cell lysis was observed even at low doses of anhydrotetracycline, suggesting that toxicity is due to an effect of mutant PcaM1 per se and not to a potential toxicity of protein overexpressed (Figures S3 and S4).

As the induction of periplasmic PcaM1 expression triggered *E. coli* cell lysis whatever the PcaM1 variant expressed, we investigated whether this phenotype was indeed the consequence of an arrest of peptidoglycan biosynthesis. Accordingly, ColM and its orthologue PaeM were previously shown to exhibit bacteriolytic and bacteriostatic effects on their specific *E. coli* and *Pseudomonas* targeted species, respectively, that were in both cases correlated to an inhibition of peptidoglycan biosynthesis [2,11]. In this respect, optical microscopy analyses showed that PcaM1-producing cells displayed greatly altered, elongated and bloated morphologies that are characteristic of an impaired cell wall biogenesis. The arrest of peptidoglycan synthesis in these cells was clearly demonstrated by (i) radiolabeling experiments, using *meso*-[^14^C]A_2_pm as a specific marker, that revealed the accumulation of the cytoplasmic UDP-MurNAc-pentapeptide precursor and the concomitant arrest of the synthesis of the polymer, as well as the presence of the lipid II degradation product; (ii) analyses of isoprenoid pool levels in membranes, which revealed the appearance and accumulation of C_55_-OH, which normally do not exist in *E. coli* cells. Quite interestingly, although the D222A PcaM1 mutant did not show detectable lipid II-degrading enzymatic activity, either in vitro (enzymatic assays) or in vivo (no C_55_-OH or 1-PP-MurNAc(pentapeptide)-GlcNAc detected in the cell content), its expression in the periplasm led to the same and specific dramatic morphological changes and arrest of peptidoglycan synthesis that have been observed with the functional wild-type PcaM1. This intriguing result could be interpreted in several ways. For instance, the D222A mutant whose lipid II hydrolase activity is abolished likely conserved its ability to interact with this substrate. A sequestration of the lipid II by the mutant protein could thus be envisaged, which would result in an inhibition of peptidoglycan polymerization steps catalyzed by the penicillin-binding proteins (PBP). Validation of such a hypothesis would need to precisely determine the relative numbers of lipid II and PcaM1 molecules present in the periplasmic space of *E. coli* in these conditions, as well as to develop in vitro analyses of these protein-substrate interactions. Further work is thus needed to elucidate by which mechanism this inactive variant of PcaM1 could still interfere so efficiently with the peptidoglycan synthesis pathway.

In this study, we also tested the periplasmic expression of the three PcaM1 variants in an *E. coli* strain carrying a deletion of the FkpA chaperone-encoding gene. Contrary to what was previously observed with periplasmic variants of ColM, i.e., that the full-length ColM protein and its isolated catalytic domain were FkpA-dependent and -independent, respectively [22], all of the PcaM1 variants constructed here were able to induce *E. coli* cell lysis in the absence of FkpA, meaning that no maturation process, at least by this chaperone, was needed for them to be toxic in *E. coli*. It thus seems that the PcaM1 protein was produced right away in an active form in these particular conditions of heterologous expression.

This work thus clearly demonstrates that the limiting reception and translocation steps usually required for colicin cytotoxic activity can be bypassed. We already knew that ColM-like orthologues were able to hydrolyze in vitro peptidoglycan lipid II intermediates of various composition and structure originating from major pathogenic bacteria (*Staphylococcus aureus*, *Enterococcus faecium, E. faecalis*) [25], and we confirmed that they could potentially do it also in vivo, as soon as they can reach the periplasmic space of the targeted bacterial species. In *E. coli*, the ColM immunity protein, Cmi (or ImM), that prevents ColM toxicity effect is located in this compartment. A gene coding for a Cmi homologue has been identified in the genome of *P. carotovorum* PC1, which likely confers immunity towards PcaM1 in this species [14]. As these enzymatic colicins clearly constitute powerful molecules with great potential as non-conventional antimicrobial agents, we now consider engineering them in order to design chimera colicins able to exhibit a broader spectrum of antibacterial activity.

## 4. Materials and Methods

### 4.1. Bacterial Strains, Plasmids and Growth Conditions

The *E. coli* strains DH5α (Bethesda Research Laboratories) and C43(DE3) (Avidis) were used as the hosts for the propagation of plasmids and the production of proteins, respectively. The *E. coli* strains FB8 and BW25113 were used for bacteriolytic activity assays, while the *E. coli* FB8 Δ*lysA*::kan was used for *meso*-[^14^C]A_2_pm incorporation experiments [24]. The *Pectobacterium carotovorum* ssp*. carotovorum* strain PC1 was kindly provided by Dr. Iris Yedidia [26]. The construction of the plasmid vector pET2160, a pET21d derivative allowing the expression of proteins with a C-terminal 6× histidine tag (His_6_), has been previously described [11]. The pREP4groESL plasmid allowing overexpression of the bacterial chaperones was obtained from Amrein, K., et al. [27]. The pASK-IBA4 vector was used for the export of proteins to the periplasm of *E. coli*, as described previously [22]. For cloning experiments, protein production and lysis experiments, cells were grown aerobically at 37 °C in 2YT medium [28], whereas they were grown in M63 minimum medium supplemented with 0.4% glucose and 100 µg/mL each of lysine, threonine and methionine for *meso*-[^14^C]A_2_pm incorporation experiments [2,24]. When needed, ampicillin and kanamycin were used at 100 and 50 µg/mL, respectively. Growth was monitored at 600 nm with a Shimadzu UV-1601 spectrophotometer.

### 4.2. Molecular Biology Techniques

Polymerase chain reaction (PCR) amplification of genes was performed in a Thermocycler 60 apparatus (Bio-Med, Guilford, CT, USA) using the Expand-Fidelity polymerase (Roche Applied Science, Indianapolis, IN, USA). DNA fragments were purified with the Wizard PCR Preps DNA purification kit (Promega, Charbonnières-les-Bains, France), and standard procedures for DNA digestion, ligation, agarose gel electrophoresis and plasmid isolations were used [29]. *E. coli* cells were transformed with plasmid DNA by the method of Dagert and Ehrlich [30] or by electroporation.

### 4.3. Construction of Expression Plasmids

A plasmid allowing high-level overproduction of the ColM homologue gene from *P. carotovorum* (*pcaM*) was constructed as follows: PCR primers Pcam-O1 and Pcam-O2 (Table 3) were designed to incorporate NcoI and BglII sites at the 5′ and 3′ extremities of the gene, respectively. The gene was amplified from the PC1 strain chromosome, and the DNA fragment was treated with NcoI and BglII and ligated between the same sites of the vector pET2160. The resulting plasmid, pMLD365, allowed the expression of the protein with a His_6_-tag (Arg-Ser-His_6_ extension) at the C-terminal extremity. To produce the D222A catalytic point mutant of PcaM1, site-directed mutagenesis of the C-terminal His_6_-tagged protein was performed directly on the pMLD365 expression plasmid by using the QuikChange II XL mutagenesis kit (Stratagene, La Jolla, CA, USA), using the pair of complementary nucleotides Pcam-mut1 and Pcam-mut2. This yielded the pMLD464 plasmid.

Export of the PcaM protein to the periplasm of *E. coli* was obtained by fusing the sequence of this bacteriocin to that of the signal peptide of OmpA, using the pASK-IBA4 plasmid as the vector. The gene was amplified with Pcam-O1 and Pcam-O3 primers, and the fragment was cleaved by NcoI and HindIII and inserted between the same sites of pASK-IBA4, yielding the pMLD381 plasmid. Plasmid pMLD395, a pMLD381 derivative plasmid expressing the D222A PcaM1 mutant, was generated by site-directed mutagenesis using the Pcam-mut1 and Pcam-mut2 oligonucleotides. Plasmid pMLD403, a pASK-IBA4 derivative plasmid expressing an N-terminally-truncated PcaM1 variant lacking the first 107 residues, was generated as described for pMLD381, except that the oligonucleotides used for PCR gene amplification were the Pcam-Δ1-107 and Pcam-O3 primers.

### 4.4. Production and Purification of Wild-Type and Mutant Forms of PcaM1

For the expression of wild-type and mutant PcaM1 proteins, C43(DE3) cells were transformed with the pMLD365 or pMLD464 plasmid, respectively, as well as with the chaperone-expressing plasmid pREP4groESL. Cells were grown at 37 °C in 2YT medium supplemented with ampicillin and kanamycin (1-liter cultures), and when the optical density at 600 nm (OD_600_) of the culture reached 0.8, isopropyl-β-d-thiogalactopyranoside (IPTG) was added at a final concentration of 1 mM and growth continued at 22 °C overnight. Then, the cells were harvested, washed with 40 mL of an 0.9% NaCl solution and finally suspended in 12 mL of Buffer A (20 mM Tris-HCl, pH 7.2, 200 mM NaCl, 0.1% 2-mercaptoethanol, 0.5 mM MgCl_2_ and 10% glycerol). In each case, the bacterial suspension was disrupted by sonication (Bioblock Vibracell sonicator, model 72412, Fisher Scientific, Illkirch, France) and then centrifuged at 4 °C for 30 min at 200,000× *g* in a TL100 Beckman centrifuge. The wild-type or mutant PcaM1-containing supernatant was subjected to purification.

His_6_-tagged wild-type and mutant forms of PcaM1 were purified first by affinity chromatography on nickel-nitrilotriacetate (Ni^2+^-NTA)-agarose polymer (Qiagen^®^, Courtaboeuf, France). All procedures were performed at 4 °C. The crude soluble supernatant obtained according to the procedure described above was mixed with 2 mL of polymer pre-equilibrated with Buffer A containing 10 mM imidazole and incubated for 30 min at 4 °C according to the Qiagen^®^ recommendations. Then, the washing and elution steps were performed with a discontinuous gradient of imidazole (20–200 mM) in Buffer A. Eluted proteins were analyzed by sodium dodecyl sulfate-polyacrylamide gel electrophoresis (SDS-PAGE). The relevant fractions were pooled, concentrated to a volume of 5 mL by centrifugation on a 10-kDa cut-off membrane (Amicon Ultra, Millipore, Molsheim, France) and then submitted to an extra purification step by gel filtration (Äkta Prime system, ^©^GE Healthcare, Buckinghamshire, UK) on a Hi-Load 16/600 Superdex S200 column (^©^GE) pre-equilibrated with one column volume of Buffer A without MgCl_2_ and glycerol and previously calibrated with blue dextran, conalbumin, ovalbumin, carbonic anhydrase, ribonuclease, aprotinine and tyrosine. Elution was performed with the same buffer at a flow rate of 1 mL/min. The purity of the fractions corresponding to the PcaM1 elution peak was checked by SDS-PAGE, and the final protein concentration was determined by using a NanoDrop^TM^ 1000 spectrophotometer (Thermo Scientific, Wilmington, DE, USA). The absorption spectra of purified wild-type and mutant forms of PcaM1 were determined using a Jasco V-630 spectrophotometer. Glycerol (10% v/v, final concentration) was eventually added for storage of the protein at −20 °C.

### 4.5. Hydrolase Activity Assays

The PcaM1 enzymatic activity was tested in a reaction mixture (10 µL) containing 100 mM Tris-HCl, pH 7.5, 20 mM MgCl_2_, 150 mM NaCl, 10 mM 2-mercaptoethanol, 12 µM [^14^C]radiolabeled lipid II (140 Bq) and 0.2% *n*-dodecyl-β-d-maltopyranoside (DDM), as previously described [11]. The reaction was started by the addition of the purified protein (in 5 µL of Buffer A) and incubated for 30 min at 37 °C with shaking (Thermomixer, Eppendorf, Wesseling-Berzdorf, Germany). For the determination of the Michaelis constants (*K*_m_), assay conditions were as described above, except that the concentration of lipid II varied from 6–100 µM. The reaction was stopped by heating at 95 °C for 1 min, and mixtures were analyzed by thin-layer chromatography (TLC) on pre-coated silica gel 60 F_254_ plates (Merck, Molsheim, France) using 1-propanol/ammonium hydroxide/water (6:3:1; v/v/v) as the mobile phase. The radioactive spots corresponding to the substrate (lipid II) and product (1-PP-MurNAc [pentapeptide]-GlcNAc) were located (*R_f_* = 0.7 and 0.3, respectively) and quantified with a radioactivity scanner (Rita Star, Raytest Isotopenmeßgeräte GmbH, Straubenhardt, Germany).

### 4.6. Bacteriolytic Activity Assays on E. coli

FB8, BW25113 and BW25113 Δ*fkpA* *E. coli* strains, carrying pASK-derived plasmids for periplasmic expression of full-length, truncated or mutant PcaM1, were grown aerobically at 37 °C in 2YT-ampicillin medium (50-mL cultures). When the OD_600_ reached 0.2, anhydrotetracycline was added at various final concentrations (from 0–400 ng/mL), and cell growth was followed by monitoring the absorbance at constant time intervals.

### 4.7. CFU

*E. coli* FB8 strains carrying the pASK plasmids allowing expression of the wild-type and D222A mutant forms of PcaM1 were grown in 2YT medium, and cultures were induced or not with 200 ng/mL of anhydrotetracycline when the OD_600_ reached 0.2. Samples were taken every 20 min and plated on 2YT agar after appropriate serial dilutions. Colonies were counted after overnight incubation at 37 °C. These experiments were performed in triplicate, for both strains and both induced and uninduced conditions.

### 4.8. Peptidoglycan Labeling Experiments with Radioactive meso-[^14^C]A_2_pm

To determine whether cells expressing wild-type or mutant forms of PcaM1 in the periplasm were truly impaired in peptidoglycan biosynthesis, the rate of incorporation of *meso*-[^14^C]A_2_pm into the peptidoglycan of *E. coli* FB8 Δ*lysA*::kan strain transformed with pASK-IBA4, pMLD381 or pMLD395 expression plasmid was followed. The latter strains were grown exponentially in minimum M63 medium (30-mL cultures) supplemented with 0.4% glucose and 100 µg/mL each of lysine, methionine and threonine [2,24]. In fact, only lysine is required to complement the *lysA*::kan mutation of these strains, but the addition of methionine and threonine was used here to decrease as much as possible the internal cellular pool of A_2_pm, as described previously [24,31]. When the OD_600_ reached 0.2, cultures were treated with anhydrotetracycline (100 ng/mL), and *meso*-[^14^C]A_2_pm (0.2 kBq/mL) was added 10 min later. The incorporation of *meso*-[^14^C]A_2_pm into the peptidoglycan polymer was then followed as described previously [2]. Briefly, 1-mL culture samples were collected regularly over time and added to 10 mL of ice-cold 5% trichloroacetic acid (TCA). Suspensions were kept on ice for 60 min, and the TCA-insoluble radiolabeled peptidoglycan material was then filtered over Whatman GF/C glass fiber filters. The filters were washed with 5% TCA and dried, and the radioactivity was counted with a liquid scintillation spectrophotometer after their immersion in a solvent system consisting of 2 mL of water and 13 mL of Unisafe 1 scintillator (Zinsser Analytic, Maidenhead, UK).

### 4.9. Cellular Distribution of meso-[^14^C]A_2_pm

To identify the step in peptidoglycan synthesis that was affected following periplasmic expression of the PcaM1 variants, the cellular distribution of [^14^C]A_2_pm incorporated in these strains was analyzed in more detail. Fifty-milliliter cultures of FB8 Δ*lysA* strain transformed with pASK-IBA4, pMLD381 or pMLD395 expression plasmids were performed as above in M63-glucose minimal medium supplemented with lysine, threonine and methionine. At an OD_600_ of 0.2, anhydrotetracycline (100 ng/mL) was added and *meso*-[^14^C]A_2_pm (0.2 kBq/mL) 20 min thereafter. After 30 min of labeling, cultures were rapidly chilled to 0–4 °C, collected by centrifugation and the cell pellets suspended in 3 mL of boiling water. After 15 min at 100 °C, suspensions were chilled and centrifuged at 200,000× *g* for 20 min. The supernatant was lyophilized, and both the soluble (supernatant) and insoluble (pellet) fractions were suspended in 250 µL of water. The total radioactivity recovered in these two cell fractions was measured. The insoluble fraction is known to contain peptidoglycan and lipid intermediates I and II, as well as the soluble fraction *meso*-[^14^C]A_2_pm and the nucleotide precursors as labeled compounds [2]. Aliquots were analyzed by TLC on silica gel plates with 1-propanol/ammonium hydroxide/water (6:3:1; v/v/v) as the mobile phase. Under such conditions, peptidoglycan remains at the origin, and UDP-MurNAc-peptides, *meso*-[^14^C]A_2_pm and lipid intermediates migrated with *R_f_* values of 0.35, 0.55 and 0.7, respectively. An additional spot (*R_f_* of 0.3) corresponding to the lipid II degradation product 1-PP-MurNAc(pentapeptide)-GlcNAc was observed following the induction of PcaM1 expression.

### 4.10. Quantitation of C_55_-P and Its Derivatives in Membranes of E. coli Strains Expressing Periplasmic PcaM1 and Derivatives

Cultures (100 mL) of *E. coli* strain FB8 expressing the different periplasmic PcaM1 variants were grown as described above, and cells were harvested just before the onset of lysis, i.e., about 40 min after the induction of protein expression by anhydrotetracycline. Isoprenoid extraction was performed from washed membrane cell pellets of the different tested strains according to two different procedures, as previously described [23]. Briefly, each culture was divided in two 50 mL samples, which were treated according to the Bligh and Dyer procedure [32] to directly quantify C_55_-P and C_55_-OH pool levels, or to Kato’s procedure [33], to determine the C_55_-PP pool level (through this procedure, C_55_-PP is totally converted into C_55_-P). The resulting isoprenoid-containing organic phases were subsequently submitted to HPLC analysis for quantitation, using an isocratic elution system (2-propanol:methanol 1:4 (v/v) containing 10 mM phosphoric acid) on a reverse-phase Nucleosil C18 column (5 µm, 250 × 4.6 mm). The flow rate was 0.6 mL/min, and the quantitation of isoprenoids, monitored at 210 nm, was performed with respect to commercial compounds previously injected as standards in the same conditions.

### 4.11. Optical Microscopy Analyses

Bacteria were visualized using a DMIRE2 optical microscope (Leica) equipped with a CCD camera (CoolSNAP HQ2, Roper Scientific, Martinsried, Germany).

### 4.12. MALDI-TOF Mass Spectrometry

MALDI-TOF mass spectra of the wild-type PcaM1 were recorded in the linear mode with delayed extraction on a PerSeptive Voyager-DE STR instrument (Applied Biosystems, Carlsbad, CA, USA) equipped with a 337-nm laser. Buffer and glycerol were removed from the samples by using a ZipTip C_4_ pipette tip (Merck Millipore, Molsheim, France) according to the manufacturer’s recommendations with slight modifications. Briefly, the bacteriocin was adsorbed on ZipTip, and after it was washed with 0.1% trifluoroacetic acid (TFA), the bacteriocin was eluted with 7.5 µL of 0.1% TFA in 70% acetonitrile. Subsequently, 1 µL of matrix solution (10 mg/mL sinapinic acid in 0.1% TFA-acetonitrile (70:30, v/v)) was deposited on the plate, followed by 0.3, 0.5 or 1 µL of concentrated bacteriocin. After evaporation of the solvents, spectra were recorded in the positive mode at an acceleration voltage of +25 kV and an extraction delay time of 300 ns. Carbonic anhydrase was used as an external calibrant.

### 4.13. Chemicals

[^14^C]lipid II labeled in the GlcNAc moiety was prepared as described previously [2]. The lipid II used in this study was C_55_-PP-MurNAc(l-Ala-γ-d-Glu-*meso*-A_2_pm-d-Ala-d-Ala)-GlcNAc, where *meso*-A_2_pm represents *meso*-diaminopimelic acid. C_55_-PP, C_55_-P and C_55_-OH were purchased from the Institute of Biochemistry and Biophysics of the Polish Academy of Sciences (Warsaw, Poland) and *meso*-[^14^C]A_2_pm from the Commissariat à l’Energie Atomique (Saclay, France). *N*-Dodecyl-*β*-d-maltopyranoside (DDM) was from Anatrace (Maumee, OH, USA), isopropyl-*β*-d-thiogalactopyranoside (IPTG) from Eurogentec (Angers, France) and Ni^2+^-nitrilotriacetate agarose from Qiagen (Courtaboeuf, France). Antibiotics and reagents were from Sigma-Aldrich (Saint-Quentin Fallavier, France). Synthesis of oligonucleotides and DNA sequencing were done by Eurofins Genomics (Ebersberg, Germany).

## Figures and Tables

**Table 1:** Pool levels of C_55_-isoprenoids in *E. coli* FB8 cells following periplasmic expression of PcaM1 and its variants.

Periplasm-Expressed Protein	Pool Level (nmol/g (Dry Weight) of Bacteria) ^a^			
	C_55_-PP	C_55_-P	C_55_-OH	Total
Control	57 ± 28	77 ± 7	ND	134 ± 31
PcaM1	0 ± 1	68 ± 10	52 ± 18	120 ± 25
Δ1-107 PcaM1	4 ± 7	80 ± 11	27 ± 12	110 ± 15
D222A PcaM1	13 ± 20	127 ± 12	ND	140 ± 20

FB8 cells carrying the pASK-IBA4 empty vector (control) or pASK plasmids expressing the wild-type PcaM1, the D222A mutant or the isolated cytotoxic domain (Δ1-107) were grown exponentially in 2YT medium, and anhydrotetracycline was added when the OD_600_ reached 0.2. Cultures were continued until an arrest of growth was observed and cells were harvested before the onset of cell lysis. The pools of isoprenoids were extracted and quantified as described in the text. ^a^ Mean ± SD of 5 independent experiments. ND, not detectable.

**Table 2:** Effects of PcaM1 variants on the cellular distribution of *meso*-[^14^C]A_2_pm incorporated into *E. coli* cells.

*E. coli* cells	Radioactivity (counts per min)		
	Soluble Fraction	Insoluble Fraction	Total
Control cells	40,240 (100%)	271,130 (100%)	311,370
+ wild-type PcaM1	168,100 (417%)	104,210 (38%)	272,310
+ D222A PcaM1	163,950 (407%)	112,420 (41%)	276,370

FB8 *lysA* cells (50 mL cultures) were grown in M63-glucose minimum medium supplemented with lysine, threonine and methionine. At an OD_600_ of 0.2, anhydrotetracycline was added and *meso*-[^14^C]A_2_pm (0.2 kBq/mL) 20 min thereafter. Cells were harvested after 30 min of labeling, just before the onset of cell lysis and were treated with boiling water. Suspensions were ultracentrifuged, and the radioactivity present in the supernatant (soluble) and pellet (insoluble) fractions was measured. The distribution of the radioactivity in these fractions (peptidoglycan and its different intermediate precursors) was then analyzed using appropriate analytical procedures, as described in the text.

**Table 3:** Oligonucleotides used in this study.

Oligonucleotides	Sequence ^a^
Pcam-O1	5’-CGCG**CCATGG**CTACTTATAAAATTAAAGATTTGACAGG-3’ (NcoI)
Pcam-O2	5’-CGCG**AGATCT**TAAACGCTGACCACGCCCAGAAATATC-3’ (BglII)
Pcam-O3	5’-CGCG**AAGCTT**ATAAACGCTGACCACGCCCAGAAATATC-3’ (HindIII)
Pcam-mut1	5’-GGATTCGTGCTTATAATGCTCTTTATGATGCCAATCCC-3’
Pcam-mut2	5’-GGGATTGGCATCATAAAGAGCATTATAAGCACGAATCC-3’
Pcam-Δ1-107	5’-CGCG**CCATGG**GATTACTTGGTGGCAACGATTCTCCAG-3’ (NcoI)

^a^ Restriction sites (in bold) introduced in oligonucleotides are indicated in parentheses, and the initiation codon of *pcaM* gene and derivatives is underlined.

## Supplementary Materials

The following are available online at www.mdpi.com/2079-6382/5/4/36/s1: Figure S1: Gel filtration elution profile and SDS-PAGE of the purified wild-type PcaM1 protein; Figure S2: MALDI-TOF mass spectrometry analysis of the purified wild-type PcaM1; Figure S3: Dose-dependent effect of anhydrotetracycline on the growth of *E. coli* FB8 cells expressing the wild-type or D222A mutant PcaM1; Figure S4: Growth and viability of *E. coli* FB8 cells expressing the wild-type or D222A mutant PcaM1; Figure S5: Effects of the periplasmic expression of the different PcaM1 variants on the growth of *E. coli* BW25113 and BW25113 *ΔfkpA* strains.

## Abbreviations

The following abbreviations are used in this manuscript:

PcaM1	pectocin M1
ColM	colicin M
Cmi	ColM immunity protein
*meso*-A_2_pm	*meso*-diaminopimelic acid
