# Supplementary material for: Pectocin M1 (PcaM1) Inhibits Escherichia coli Cell Growth and Peptidoglycan Biosynthesis through Periplasmic Expression
Source: Antibiotics (Basel). 2016 Oct 8;5(4):36. doi: 10.3390/antibiotics5040036 (PMC5187517; doi:10.3390/antibiotics5040036)
Supplement: Supplementary file 1 [file antibiotics-05-00036-s001.pdf]

Pectocin M1 (PcaM1) Inhibits *Escherichia coli* Cell Growth and Peptidoglycan Biosynthesis through Periplasmic Expression

Dimitri Chérier, Sean Giacomucci, Delphine Patin, Ahmed Bouhss, Thierry Touzé, Didier Blanot, Dominique Mengin-Lecreulx and Hélène Barreteau

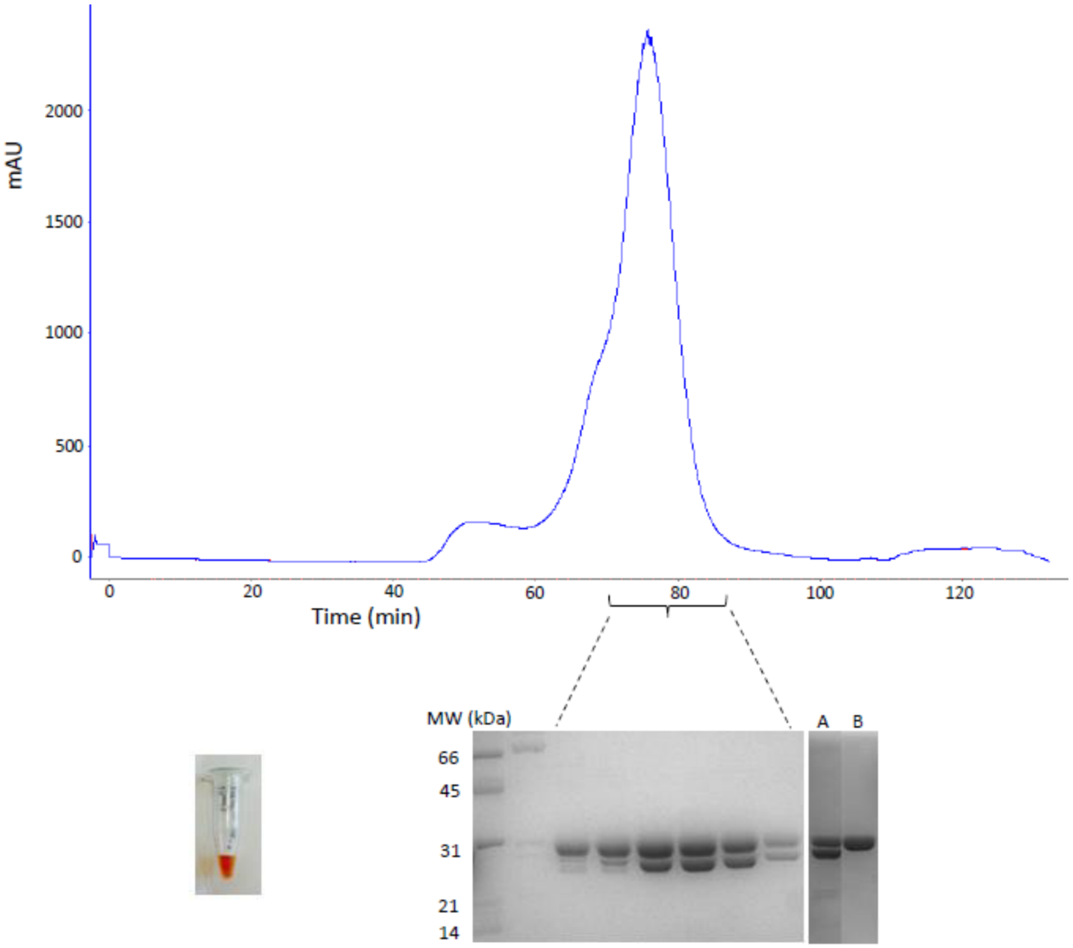

Figure S1. Gel filtration elution profile and SDS-PAGE of the purified wild-type PcaM1 protein. The absorbance at 280 nm was monitored, 2 mL fractions eluted between 72 and 84 min, and corresponding to the homogenous PcaM1, were collected and loaded onto SDS-PAGE. Additional lanes A and B represent native and boiled PcaM1 samples, respectively. As shown on the left (Eppendorf tube, Wesseling-Berzdorf, Germany), the purified concentrated PcaM1 has a red-brown color.

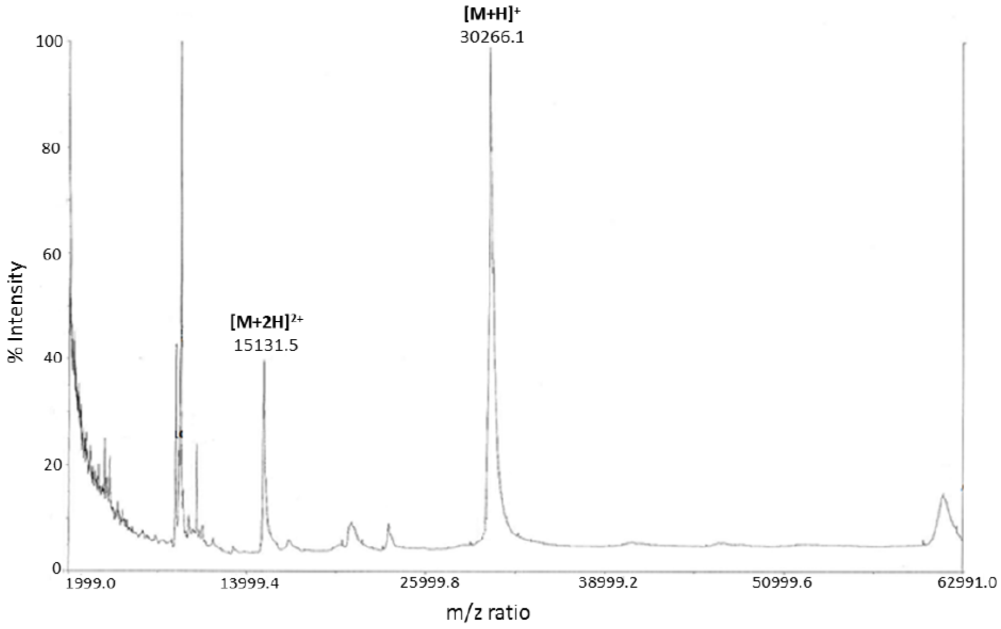

Figure S2. MALDI-TOF mass spectrometry analysis of the purified wild-type PcaM1. Peaks of *m*/*z* 30266.1 and 15131.5 were observed, that were assigned to be the $[M+H]^+$ and $[M+2H]^{2+}$ ions, respectively.

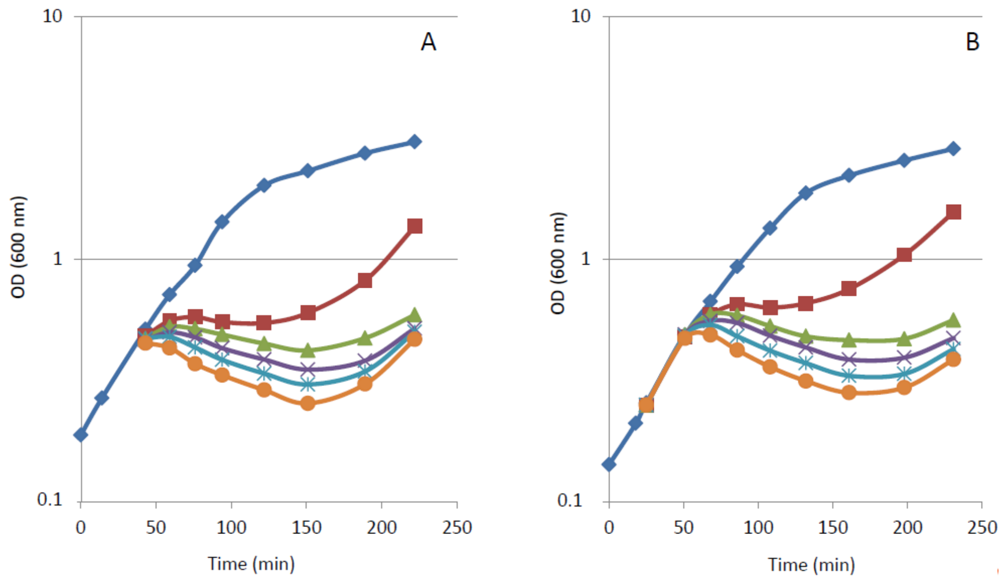

Figure S3. Dose-dependent effect of anhydrotetracycline on the growth of *E. coli* FB8 cells expressing the wild-type (**A**) or D222A mutant (**B**) PcaM1. Cells were grown at 37 °C in 2YT medium and protein expression was induced by addition of anhydrotetracycline at OD_{600} = 0.25 (t_0). Diamonds, squares, triangles, crosses, stars and circles correspond to anhydrotetracycline concentrations of 0, 30, 45, 60, 100 and 200 ng/mL, respectively.

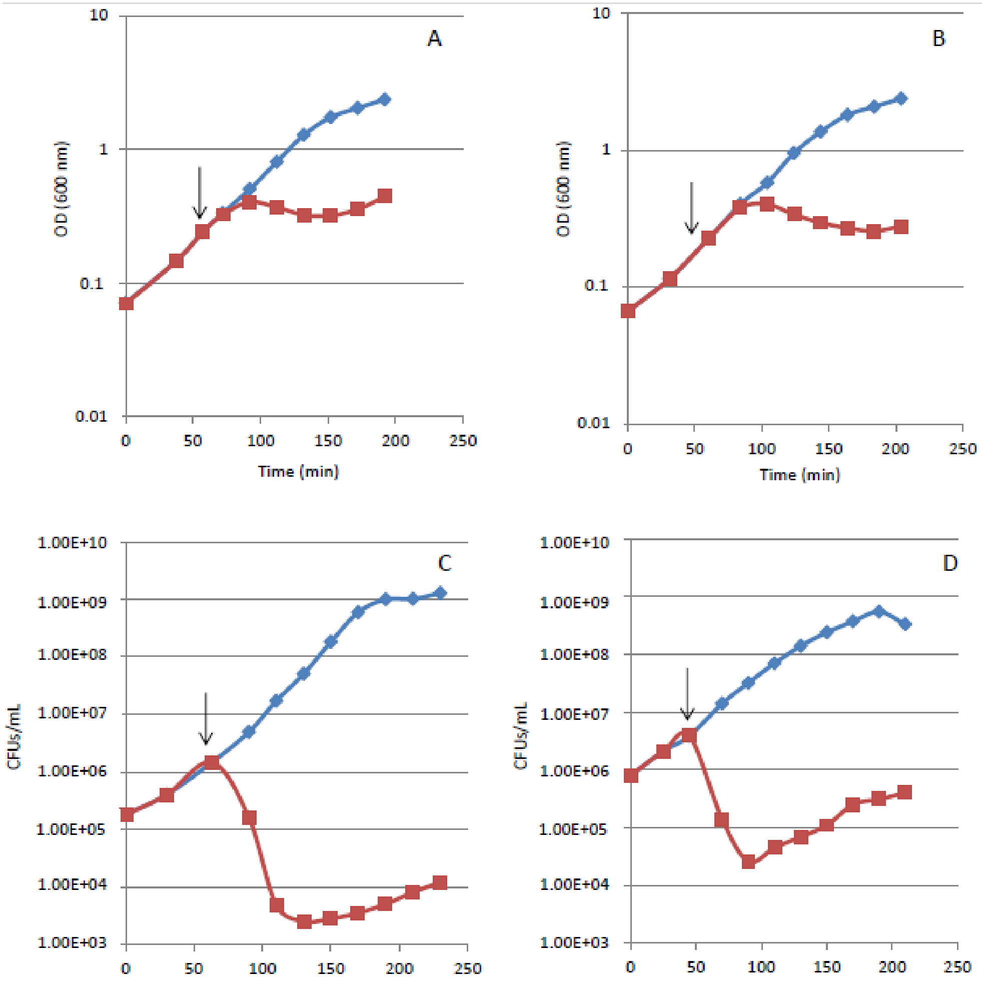

Figure S4. Growth (**A**,**B**) and viability (**C**,**D**) of E. coli FB8 cells expressing the wild-type (**A**,**C**) or D222A mutant (**B**,**D**) PcaM1. Strains were grown in 2YT medium at 37 °C. At OD_{600} = 0.25, anhydrotetracycline (arrows) was added (red curves) or not (blue curves) at a final concentration of 200 ng/mL. The representative results of three independent experiments are presented.

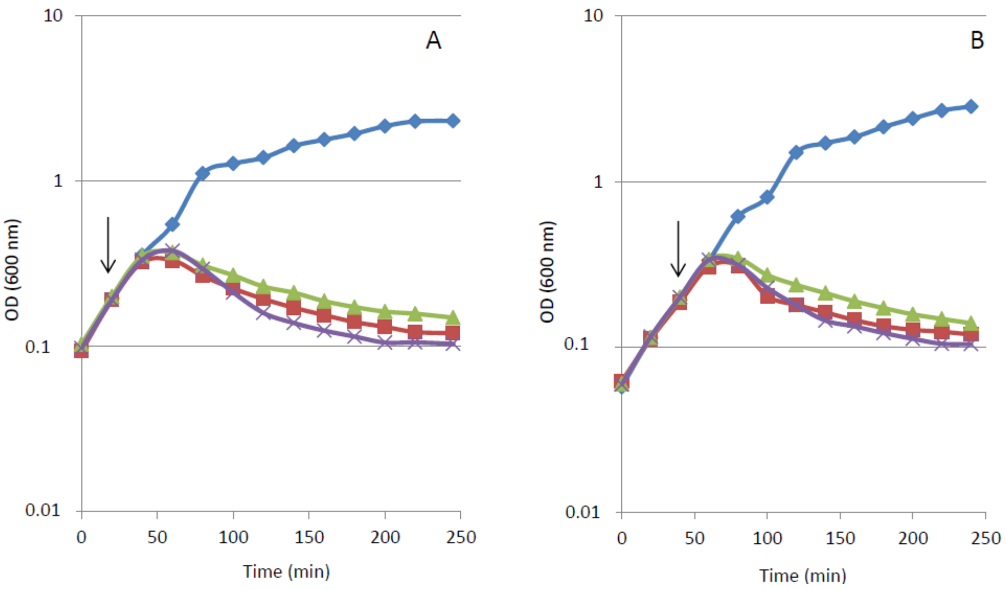

Figure S5. Effects of the periplasmic expression of the different PcaM1 variants on the growth of *E. coli* BW25113 (**A**) and BW25113 *ΔfkpA* (**B**) strains. Cells were grown in 2YT medium at 37 °C and expression of the PcaM1 variants was induced by addition of anhydrotetracycline at 200 ng/mL (arrows). Growth curves observed for strains carrying either the pASK-IBA4 empty vector, or pASK plasmids expressing the wild-type PcaM1, the D222A mutant or the isolated cytotoxic domain (Δ1-107) are shown by diamonds, squares, triangles and crosses, respectively. The representative curves of three independent experiments are presented.

© 2016 by the authors; licensee MDPI, Basel, Switzerland. This article is an open access article distributed under the terms and conditions of the Creative Commons by Attribution (CC-BY) license (http://creativecommons.org/licenses/by/4.0/).
